# *Gilsonicaris* from the Lower Devonian Hunsrück slate is a eunicidan annelid and not the oldest crown anostracan crustacean

**DOI:** 10.1098/rsbl.2023.0312

**Published:** 2023-08-30

**Authors:** P. Gueriau, L. A. Parry, N. Rabet

**Affiliations:** ^1^ Université Paris-Saclay, CNRS, ministère de la Culture, UVSQ, MNHN, Institut photonique d'analyse non-destructive européen des matériaux anciens, 91192 Saint-Aubin, France; ^2^ Institute of Earth Sciences, University of Lausanne, Lausanne, Switzerland; ^3^ Centre for Life's Origins and Evolution, Department of Genetics, Evolution and Environment, University College London, London, UK; ^4^ BOREA, Sorbonne Université, Muséum national d'Histoire naturelle, Paris, France

**Keywords:** Branchiopoda, Polychaeta, scolecodonts, soft-tissue, X-ray microtomography, Early Devonian

## Abstract

The Lower Devonian (Lower Emsian, −400 Myr) roof slates of the Hunsrück in southeastern Germany have delivered a highly diverse and exceptionally preserved marine fauna that provides a unique snapshot into the anatomy and ecology of a wide range of Palaeozoic animals. Several of the described taxa, however, remain enigmatic in their affinity, at least until new pyritized features hidden under the surface of the slate are revealed using X-ray radiography or micro-computed tomography (µCT). Here, we redescribe such an enigmatic fossil, the putative anostracan crustacean *Gilsonicaris rhenanus* Van Straelen, 1943. Using µCT scanning, we unveil unprecedented details of its anatomy, including a ventral oral opening and four pairs of recalcitrant jaw elements. These jaws are morphologically consistent with the scolecodonts of eunicidan polychaetes, which along with the gross anatomy of the body and head unambiguously identifies *G. rhenanus* as a polychaete rather than an arthropod. While this discovery firmly discards the Early Devonian record of crown anostracans in the fossil record, it adds a new record of eunicidan soft tissues, which are surprisingly rare considering the abundant microfossil record of scolecodonts.

## 1. Introduction

**Figure 1.**
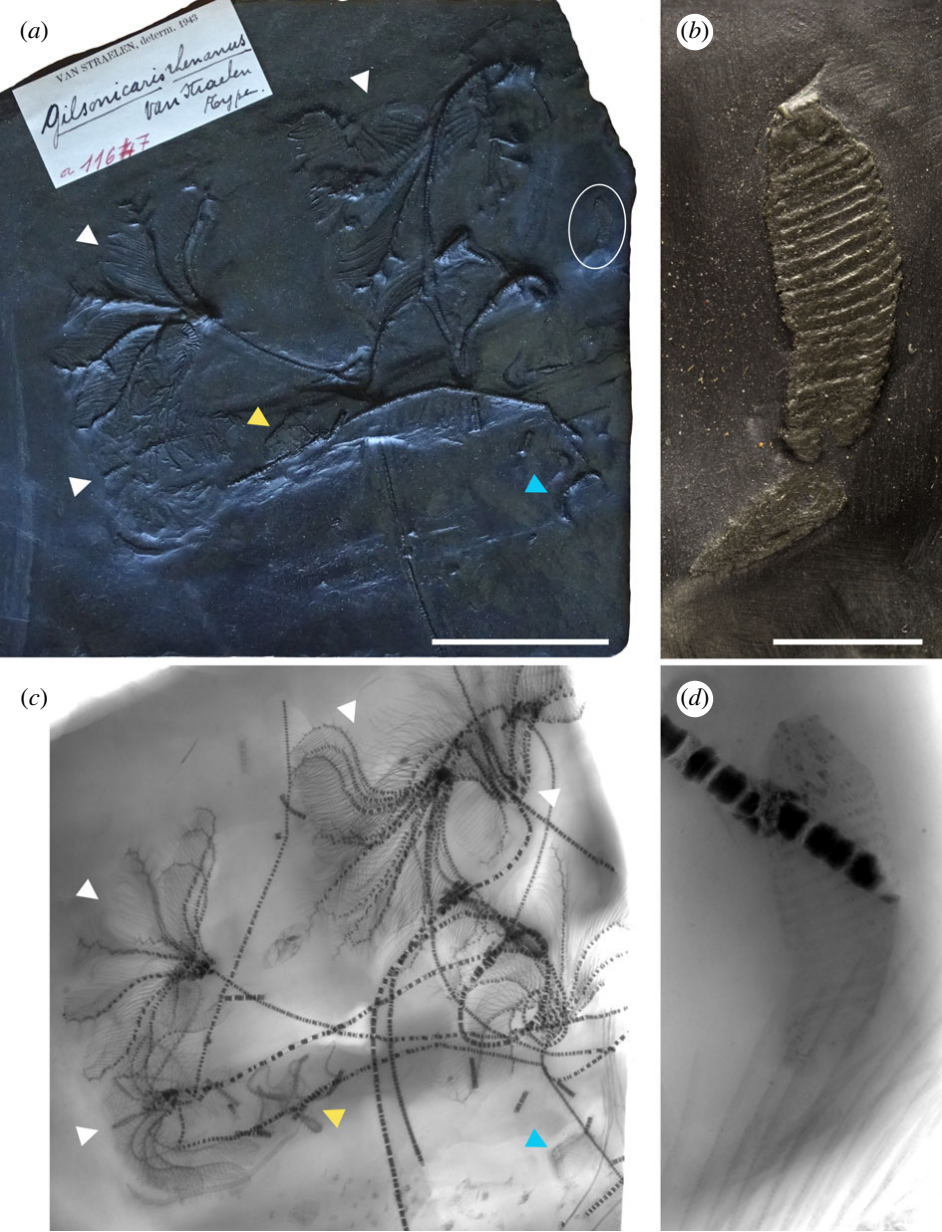
*Gilsonicaris rhenanus* Van Straelen, 1943, holotype IRSNB a11647. (*a*,*c*) Optical photograph (*a*) and X-ray radiograph (*c*) of the slate; the ellipse in (*a*) highlights the position of *G. rhenanus*. Note that the slate is rich in other fossils, including notably numerous crinoids (white arrowheads), as well as an asterozoan echinoderm (yellow arrowheads) and a rugose coral (blue arrowheads) nicely revealed by X-ray radiography. (*b*,*d*) Close-up photograph (*b*) and radiograph (*d*) of *G. rhenanus*. Scale bars = 5 cm (*a*,*c*), 5 mm (*b*,*d*).

**Figure 2.**
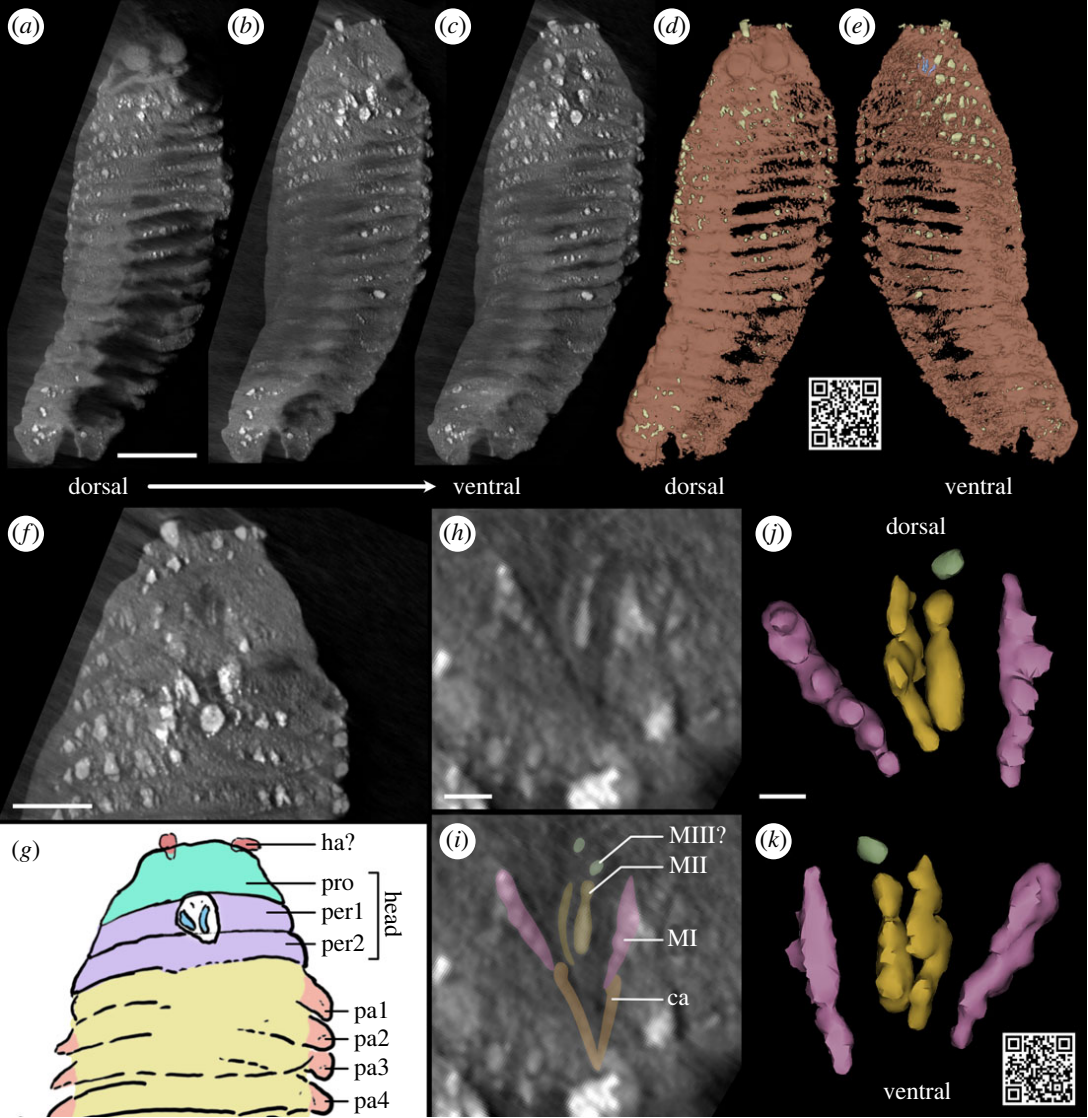
X-ray micro-computed tomography of *Gilsonicaris rhenanus* Van Straelen, 1943, holotype IRSNB a11647. (*a–c*) Dorsal (*a*), central (*b*) and ventral (*c*) coronal tomographic slices through the fossil. (*d*,*e*) Three-dimensional rendering of the fossil in dorsal (*d*) and ventral (*e*) views. (*f*,*g*) Close up (*f*) and interpretative drawing (*g*) of the anterior region from a slice slightly more ventral than (*b*). (*h*,*i*) Close up (*h*) and interpretative drawing (*i*) of the jaws from (*b*). (*j*,*k*) Three-dimensional rendering of the jaws in dorsal (*j*) and ventral (*k*) views. The displayed QR codes direct towards the three-dimensional models shared on Sketchfab. Abbreviations: ca, carriers; ha?, possible head appendages; MI–III, maxillary elements I–III; pa1–4, parapodia 1–4; per1–2, peristomium ring 1–2; pro, prostomium. Scale bars = 2 mm (*a–e*), 1 mm (*f*,*g)*, 200 µm (*h,i*), 100 µm (*j*,*k*).

*Gilsonicaris rhenanus* Van Straelen, 1943 (figure 1*a,b*) is a 16-mm-long segmented organism from the Lower Devonian (Lower Emsian) Hunsrück Slate (southeastern Germany), known by a single specimen, originally described as an anostracan crustacean (fairy shrimp) based upon the identification of a cephalon followed by 11 segments bearing appendages and at least 18 segments without appendages [1]. This fossil is only slightly younger than the stem-group anostracan *Lepidocaris rhyniensis* Scourfield, 1926 [2] from the Lower Devonian (Pragian) Rhynie Chert (Scotland), suggesting the presence of more modern-looking anostracans as early as during the Emsian. Anostracans then have no fossil record until the Upper Devonian (Famennian) channel filling deposits of Strud (Belgium) [3]. Nonetheless, significant doubts have been cast on the affinities of *Gilsonicaris*. Rolfe [4] proposed an alternative interpretation as a possible juvenile of the arthropleurid myriapod *Bundenbachiellus minor* Broili, 1930, a suggestion that has not been followed up by later work on *Bundenbachiellus*. The most recent works on fossil anostracans do not recognize *Gilsonicaris* as an anostracan, because it does not possess any anostracan synapomorphies beyond a possibly homonymous trunk [5,6]. It has even been postulated that it is perhaps not even an arthropod but part of an asteroid echinoderm arm (see [5]). Here, we re-evaluate the affinities of *Gilsonicaris* using X-ray micro-computed tomography (µCT, figure 2), which unveils four pairs of internal jaw elements (scolecodonts) distinctive of polychaete annelids.

## 2. Material and methods

### (a) Specimen

*Gilsonicaris rhenanus* Van Straelen, 1943 is known from a single specimen, housed at the Royal Belgian Institute of Natural Sciences (Brussels, Belgium) under the accession number IRSNB a11647. The fossil was recovered during the mining of the Lower Devonian Hunsrück roof slates in Bundenbach, but more precise provenance was not given. The specimen was photographed using a Canon EOS 800D SLR camera equipped with a Canon MP-E 65-mm macro lens. Image stacking was used to combine photographs collected at differing focal planes into a composite with enhanced depth of field, using Adobe Photoshop.

### (b) X-ray imaging

X-ray radiography of the slate and µCT of *Gilsonicaris* were performed at the AST-RX imaging platform of the Muséum national d'Histoire naturelle (Paris, France), using a GE Sensing and an Inspection Technologies phoenix | X-ray v | tome | ×L240-180 CT scanner. Van Straelen [1] described and figured segmentation on the disarticulated posterior part of the specimen, a feature that is not clearly observed today on the specimen, neither using optical microscopy (figure 1*b*) nor using X-ray radiography (figure 1*d*). We therefore limited µCT scanning to the articulated anterior part of the specimen, which also enabled higher resolution to be achieved. In total, 2600 projections were collected over 214° to account for the extremely flat nature of the specimen, using three averaged images per projection, 1 s of exposure time and one skipped image before each projection. Voltage and current were set to 90 kV and 300 mA, respectively. The volume was reconstructed using the phoenix datos| xfi 2.0 reconstruction software, yielding an isotropic voxel size of 10.15 µm. The dataset was then exported into an 8-bit TIFF image stack of 48 coronal slices (the dataset (29 MB) is available on Morphosource; https://www.morphosource.org/concern/media/000523565). Segmentation and three-dimensional rendering were performed using the software MIMICS Innovation Suite 19.0 (Materialize) at the IPANEMA laboratory. Automatic thresholding and manual selection were used for the segmentation of the cuticle and mouthparts, respectively. µCT-derived density differences between distinctive materials were assessed qualitatively in ImageJ by extracting grey-value histograms from areas of interest using the freehand selection tool (electronic supplementary material, figure S1).

## 3. Results and discussion

### (a) *Gilsonicaris* is not an anostracan crustacean

Three-dimensional observation using µCT data (figure 2) reveals the absence of articulated folacious appendages and a well-distinct head with pedunculated eyes. Instead, clear morphological similarities with polychaete annelids can be identified, such as many segmented body lateral outgrowths/appendages (parapodia), a head with differentiated prostomium and peristomium formed of two rings (which houses a ventral mouth) and a multi-element jaw apparatus that is comparable with that of eunicidan polychaetes. µCT has so far been applied only on rare occasions to Hunsrück fossils, firstly to an machaeridian annelid [7] and later to two arthropods, a vertebrate, a mollusc and an echinoderm [8–12]. Considering the level of detail and information it revealed for such a limited but wide range of organisms, including specimens as small and flattened as *Gilsonicaris*, our data further highlights the potential of µCT for uncovering new insights into the systematics and palaeoecology of the Hunsrück fauna, and also by extension into mid-Palaeozoic marine ecosystems.

### (b) Detailed description of the specimen

The body is externally annulated on both the dorsal (figures 1*d* and 2*d*) and ventral surface (figure 2*e*) and is partitioned into a clearly defined head region and trunk with small lateral outgrowths that are most clearly visible in the anterior region of the right side of the trunk. In total, approximately 17 trunk segments can be identified (see below for the composition of the head). The body appendages project laterally, approximately 500 µm from the lateral body wall. Segments are 50–200 µm thick, but can locally reach 300–330 µm where undercoated by large, most likely pyrite, crystals (electronic supplementary material, figure S2). This substantial dorsoventral flattening, together with relatively coarse preservation and preparation dorsally, precludes the identification of fine scale details, such as chaetae, cirri, branchiae or differentiated rami. The posterior region of the body is incomplete and consequently the total body length is unknown.

The head consists of a rounded prostomial lobe *ca* 800 µm in length, there are no appendages that can be easily identified, except for two small (approx. 300 µm) projections placed ventro-laterally on the anterior margin of the head. The prostomium is followed by two segment-like units that are approximately 2.4 mm wide with no identifiable appendages (figure 2*f,g*). These structures and following segments are approximately 400 µm in length that broaden to approximately 3.4 mm at the ninth segment. The three-dimensional reconstruction reveals that ventrally these units contain the mouth region (figure 2*f*), identifying them as peristomial rings, rather than appendageless anterior segments.

Internally, the oral region contains a jaw apparatus that consists of at least four bilateral pairs of elements (figure 2*h,i*), that are arranged either side of the midline of the body. The density of the jaws is intermediate between cuticular remains and probable pyrite crystals (electronic supplementary material, figure S1) potentially indicating some degree of mineralization in life, or retention of metal ions chelated to the jaw tips, as is widespread in annelid jaws [13]. Each maxillary element is 80–100 µm in diameter. The pair of elements closest to the body midline (MII from here onwards, see discussion for reasoning behind the identification of different elements) are slightly shorter (350 µm long) than the more lateral (MI, see discussion) elements that are approximately 420 µm long. There is no pronounced difference in size of the corresponding elements on either side of the body, as is widespread in many eunicidan polychaetes, e.g. Oenonidae [14], although the right MI is slightly shorter than the corresponding left element. Both elements possess prominent dorsally projecting denticles, with six denticles visible on the MI elements that range from 40 to 65 µm in diameter. These denticles are prominent in the three-dimensional models, but are also clearly visible as brighter regions in the tomography slices (figure 1*h,i*). The dorsal ornament of the MII elements is less clear, although the left MII may possess four or five discrete denticles. In addition, there is a smaller element displaced anteriorly from the right MII element, which most likely represents a right MIII element (see discussion). Posterior of the maxillary elements is a pair of structures that resembles the carriers of eunicidan jaws, but these are not sufficiently distinct from the surrounding material to be manually segmented with accuracy (figure 2*h,i*). Their indistinct preservation is likely a consequence of the thinness of these structures in life. Likewise, there are features visible in the µCT slices that represent additional maxillary elements (figure 2*h,i*), including a left MIII element. As for the carriers, they cannot be extracted from the µCT slices with precision as they are composed of few voxels.

### (c) Taxonomic assignment and implications

Among annelids, robust jaw elements are found within the errant groups Eunicida and Phyllodocida but are also found in members of Sedentaria, namely in Ampharetidae [15] and some leeches, i.e. Arynchobdellida [16]. The jaws of both taxa do not resemble the condition observed in *Gilsonicaris,* and neither do they resemble it in gross morphology and so are not considered further here. Errant polychaetes produce a diversity of jaw structures that most likely have multiple, independent and ancient origins, with the oldest jawed eunicidans occurring in the Cambrian and diversifying in the Ordovician [17]. While jawed phyllodocidans are first known in the Ordovician, the major jawed lineages are not otherwise identified from body and microfossils until the Carboniferous, by which time at least the total groups of all of the jaw producing lineages have been identified [18,19], although the precise taxonomic assignments of some of these taxa remain unclear [20].

While the coarse preservation of the jaws precludes a detailed comparison with extant taxa and the rich fossil record of jawed polychaetes, their gross morphology and arrangement provide sufficient information to identify *Gilsonicaris* as a member of Eunicida to the exclusion of other alternatives. Phyllodocidans either possess a bilateral pair of elements in ‘nereidiforms' (Chrysopetalidae, Nereididae, Hesionidae and Nephtyidae), a pair of elements with associated, self-similar micrognaths or four jaws in a dorsoventrally biting pairs (Aphroditiformia) or in a ring (Glyceridae), see Parry *et al*. [21]. Eunicidans possess a complex jaw apparatus with paired left and right elements [14], consisting of a ventral pair mandibles, and dorsal maxillae. In addition, there is typically a posterior pair of carriers that aid in movement of the maxillae, although these are absent in the most ancient fossils and the early diverging group Dorvilleidae [14]. Corresponding maxillae of each side are typically not mirror images, and in many clades (e.g. Oenonidae) corresponding left and right elements differ strongly in both size and shape [14].

Paxton [14] defined six different apparatus types, of which four possess carriers: labidognath, eulabidognath, prionognath and symmetrognath. In the first three, the apparatuses are strongly asymmetrical, and the right MI element has been reduced to a ‘basal plate' [14], which is not observed in *Gilsonicaris*, whose jaw apparatus resembles those of the symmetrognath type, given its sub-symmetrical morphology. Note that we follow the terminology of Paxton [14], and regard the basal plate/laeobasal plate of symmetrognaths (and consequently *Gilsonicaris*) as homologous to the first maxillary elements of other eunicidan taxa, and refer to them as MI throughout. This grade of jaw apparatus is known from two extant families, Hartmaniellidae and Lumbrineridae, of which only the former has a known fossil record, and two extinct families, Conjugaspidae and Symmetroprionidae [14,22,23]. In both *Conjugaspis* and *Symmetroprion*, the denticles of the MI elements (=basal/laeobasal plates in [22,24]) project laterally, rather than dorsally and they are more numerous than in *Gilsonicaris* (greater than 10) on both the MI element and the MII elements, although the denticles of MII project dorsally, as in *Gilsonicaris*. The maxillae anterior to the MII elements in both taxa are unknown [22,24], suggesting that they are either absent, or relatively small and indistinct, as in *Gilsonicaris*. The jaws of Lumbrineridae and Hartmaniellidae are closer in morphology to that of *Gilsonicaris* as they possess fewer denticles, although the MI element of lumbrinerids lacks denticles [14] and the MI denticles are laterally (rather than dorsally) orientated in extant and extinct hartmaniellids [25]. The relative size of the MI and MII elements varies between taxa with symmetrical jaw apparatuses.

The polychaete taxa with sub-symmetrical jaw apparatuses have previously been considered a clade based on their similar jaw architecture [14], but phylogenomic data are currently lacking for Hartmaniellidae, so this hypothesis is yet to be confirmed based on data independent of jaw morphology. Nevertheless, given the gross similarities in jaws to other extant and extinct symmetrognaths, we tentatively assign this taxon to the total group of symmetrognaths. This assignment within Eunicida is not contradicted by the limited insights into the soft anatomy. Within Eunicida, the peristomium is formed of two rings in all families except Onuphidae (where it is a single ring) and the prostomium forms a prominent lobe that is often rounded in morphology like that of *Gilsonicaris,* particularly in Oenonidae and Lumbrinderidae [26]. The identity of the anterior projections is not clear, and this is not resolved by a comparison with extant symmetrognaths, as both *Hartmaniella* and Lumbrineridae lack antennae and palps altogether. Nevertheless, other eunicidans have median and lateral antennae (e.g. Oenonidae), lateral antennae and a pair of palps (Dorvilleidae) or a median antenna, lateral antennae and a pair of palps (Eunicidae and Onuphidae) suggesting that symmetrognath taxa have lost a total of five head appendages as these appendages are all present in Phyllodocida. Of the eunicidans that possess palps, they are antero-lateral in Onuphidae and Euncidae or ventral in Dorvilleidae, whereas antennae are dorsally placed in the eunicidans that possess them. The anterolateral appendages of *Gilsonicaris* are therefore most likely palps with a comparable position to those of Onuphidae and Eunicidae, therefore potentially shedding light on the sensory apparatus of early symmetrognaths, suggesting that this group retained palps at least until the Devonian.

Six polychaete annelid genera (including a machaeridian [7]) have previously been described from the Hunsrück Slate, with *Ewaldips feyi* [27] most closely resembling *Gilsonicaris.* Nevertheless, further anatomical comparison requires re-investigation of the polychaete fauna using µCT as these taxa have only been investigated using X-ray radiography thus far. While this did reveal a wealth of features that are otherwise non-visible (as is also the case for *Gilsonicaris*; figure 1*d*), µCT has great potential to reveal further anatomical detail, as illustrated herein for *Gilsonicaris* (figure 2).

## 4. Conclusion

Our redescription of the single known specimen of *Gilsonicaris rhenanus* Van Straelen, 1943 using state-of-the-art X-ray µCT scanning provides unambiguous evidence that it is not an anostracan crustacean or even an arthropod but a polychaete annelid. Newly revealed anatomical details include the differentiation of the head into a prostomium and a peristomium formed of two rings, a ventral mouth and a multi-element jaw apparatus typical of eunicidan polychaetes, the morphology of which suggest affinities with symmetrognaths. Altogether, these findings firmly discard the only Early Devonian record of crown anostracans, enrich the fossil record of eunicidan taxa and their soft tissues, documenting in particular a retention of palps at least until the Devonian in symmetrognaths, and call for a re-investigation of the Hunsrück polychaete fauna using µCT scanning.

## Data Availability

Requests for access to the fossil specimen should be addressed to Annelise Folie (afolie@naturalsciences.be) of the Royal Belgian Institute of Natural Sciences (Brussels, Belgium). The µCT dataset (tomographic slices) and three-dimensional models generated in this study are available on MorphoSource (https://www.morphosource.org/concern/media/000523565) and Sketchfab (https://skfb.ly/ow8w7; https://skfb.ly/ow8xH), respectively. Data and R script used for the qualitative assessment of the density of the different materials present in the fossil (electronic supplementary material, figure S1) are available via the Dryad Digital Repository: https://doi.org/10.5061/dryad.rxwdbrvfn [28]. Figure S1 and S2 are provided in the electronic supplementary material [29].
